# Cohort profile of the South London and Maudsley NHS Foundation Trust Biomedical Research Centre (SLaM BRC) Case Register: current status and recent enhancement of an Electronic Mental Health Record-derived data resource

**DOI:** 10.1136/bmjopen-2015-008721

**Published:** 2016-03-01

**Authors:** Gayan Perera, Matthew Broadbent, Felicity Callard, Chin-Kuo Chang, Johnny Downs, Rina Dutta, Andrea Fernandes, Richard D Hayes, Max Henderson, Richard Jackson, Amelia Jewell, Giouliana Kadra, Ryan Little, Megan Pritchard, Hitesh Shetty, Alex Tulloch, Robert Stewart

**Affiliations:** 1 King's College London (Institute of Psychiatry, Psychology and Neuroscience), London, UK; 2 South London and Maudsley NHS Foundation Trust, London, UK; 3 Durham University, Durham, UK

**Keywords:** BIOTECHNOLOGY & BIOINFORMATICS, EPIDEMIOLOGY, MENTAL HEALTH, PSYCHIATRY

## Abstract

**Purpose:**

The South London and Maudsley National Health Service (NHS) Foundation Trust Biomedical Research Centre (SLaM BRC) Case Register and its Clinical Record Interactive Search (CRIS) application were developed in 2008, generating a research repository of real-time, anonymised, structured and open-text data derived from the electronic health record system used by SLaM, a large mental healthcare provider in southeast London. In this paper, we update this register's descriptive data, and describe the substantial expansion and extension of the data resource since its original development.

**Participants:**

Descriptive data were generated from the SLaM BRC Case Register on 31 December 2014. Currently, there are over 250 000 patient records accessed through CRIS.

**Findings to date:**

Since 2008, the most significant developments in the SLaM BRC Case Register have been the introduction of natural language processing to extract structured data from open-text fields, linkages to external sources of data, and the addition of a parallel relational database (Structured Query Language) output. Natural language processing applications to date have brought in new and hitherto inaccessible data on cognitive function, education, social care receipt, smoking, diagnostic statements and pharmacotherapy. In addition, through external data linkages, large volumes of supplementary information have been accessed on mortality, hospital attendances and cancer registrations.

**Future plans:**

Coupled with robust data security and governance structures, electronic health records provide potentially transformative information on mental disorders and outcomes in routine clinical care. The SLaM BRC Case Register continues to grow as a database, with approximately 20 000 new cases added each year, in addition to extension of follow-up for existing cases. Data linkages and natural language processing present important opportunities to enhance this type of research resource further, achieving both volume and depth of data. However, research projects still need to be carefully tailored, so that they take into account the nature and quality of the source information.

**Strengths and limitations of this study**

- Because the Clinical Record Interactive Search (CRIS) model draws directly from the electronic health record, it provides valuable ‘real-world’ and ‘real-time’ information on routine mental healthcare, automatically accumulating large volumes of data without any requirement for service reconfiguration or changes at the clinical interface.
- Although electronic health records-based registers remove the requirement for specific ‘data collection’ in routine clinical care, a major challenge for mental health data in particular is that most information is recorded in text rather than structured fields. Natural language processing offers important opportunities for data enhancement.
- External data linkages are also potentially valuable, but dependent on the nature of the data supplemented—most often providing additional information on exposures and outcomes outside mental health domains and between care episodes rather than on the nature of mental disorders themselves.
- Regardless of the volume of data available, it is important to bear in mind their provenance (ie, highly dependent on what information a clinical staff member records or not); research applications need to be tailored with this in mind.
- A key challenge inherent with all use of healthcare data is data protection, and it is important to develop anonymised data resources in a way that is acceptable to the general public, and to the patients whose personal and often highly sensitive information forms the database. Such challenges incorporate not only a case register's data themselves but also procedures around data linkage where use of identifiers is required.

## Introduction

It is nearly 30 years since the publication of Ten Horn *et al*'s^1^ comprehensive inventory of the psychiatric case register and its use in research. Seven years ago electronic health record (EHR)-based registers were proposed as a possible ‘new generation’.^2^ The longitudinal nature of case registers, their size and coverage of defined populations make them an important research asset, providing large numbers of participants and measurement points, as well as the potential for data linkage.^3^ Recent years have seen an increase in the use of the psychiatric case register for research purposes, including linkage across diverse health and other population databases, including criminological information resources.^4^ There are several unique applications of case registers. Despite the methodological advantages of the randomised controlled trial, observational data remain fundamental to health research, and much of what we know (or assume we know) is derived from observation rather than experimental intervention.^5^ Although they can contribute to aetiological research, case registers are particularly suited to the investigation of the course and outcome of a disorder, as well as allowing intervention response to be evaluated in large, naturalistic samples and settings. In smaller scale psychiatric case registers, quality of data can be more regularly checked and the number of variables collected can be higher than in a large database. These registers can include information on the clinical condition of the patients, on psychopharmacological treatments and on duration of contacts.^6^ The combination of quality and quantity in data renders small-scale registers of great interest for researchers and policymakers. EHRs in mental healthcare, on the other hand, represent data which are potentially both large and deep—because in theory, these contain every piece of information that has been recorded in a clinical service about a person’s presentation, symptoms and relevant background history, as well as interventions received and observed outcomes.^5^

Through technological advances in both the daily updating and validation of registers, large and complex projects can be carried out. Register data are particularly suited to supporting comprehensive longitudinal studies of the course of illness to predict outcomes and naturalistic response to interventions. With EHRs increasingly complementing or replacing handwritten notes in mental health services, large volumes of clinical information are now already contained in an electronic format. This removes the requirement for de novo data collection and entry which presented formidable challenges for earlier registers, albeit processes with a higher potential for quality control. Local EHR-sourced registers are more likely to be limited by migration between geographic catchments, but their strength lies in their ability to cover all types of service within a given area, thereby providing a more comprehensive picture of mental health than is afforded by national registers.

The South London and Maudsley National Health Service (NHS) Foundation Trust Biomedical Research Centre (SLaM BRC) Case Register was set up in 2008 as a novel data resource derived directly from the routine EHRs of a large mental healthcare provider, and its initial development was outlined in 2009.^7^ At the time of analysis for that paper (October 2008), the database contained 123 000 cases and information available through the Clinical Record Interactive Search (CRIS) application was primarily restricted to that imposed by the format of the source EHR fields. Since then, the SLaM BRC Case Register has expanded substantially, not only in case numbers (now over 250 000) but also, most importantly, in the scale and depth of derived and externally linked information available. The objective of this paper is to update the description of this case register and, particularly, to outline technical developments which have enhanced the depth of information available, and which we believe have potential generalisability to other comparable clinical data resources.

## Cohort description

### The SLaM BRC Case Register and CRIS application

Initial development of the SLaM BRC Case Register has been previously described in detail, as has SLaM as a provider (and see also http://www.slam.nhs.uk).^7^ In summary, the data are sourced from EHRs used by SLaM, which provides comprehensive mental health services to a geographic catchment of over 1.2 million residents in four south London boroughs—Croydon, Lambeth, Lewisham and Southwark—as well as some regional/national specialist services. SLaM catchment service provision is currently structured within the following specialty groupings: Addictions; Behavioural and Developmental Psychiatry; Child and Adolescent Mental Health Services; Mental Health of Older Adults and Dementia; Mood, Anxiety and Personality; Psychological Medicine; Psychosis. These are aligned with academic groupings at King's College London, reflecting the university–health service partnership enshrined within King's Health Partners Academic Health Sciences Centre (KHP AHSC; http://www.kingshealthpartners.org; also incorporating two major acute care providers). The groupings also encompass services delivered to all age groups, standard specialties such as Addictions, Eating Disorders and Learning Disabilities, as well as provision within Forensic and General Hospital Liaison settings. In addition, wider national provision by SLaM at the time of writing includes the following services: adult attention deficit hyperactivity disorder, adult personality disorder, affective disorders, anxiety disorders (residential), autism assessment and behavioural genetics, brain injury (outpatient and inpatient), anxiety disorders and trauma, chronic fatigue, eating disorders (day care, outpatients, inpatients), female hormone clinic, psychosis (inpatient, outpatient and specialist rehabilitation), mother and baby unit, autism, practitioner health, psychological interventions, psychosexual disorders, self-harm (outpatients) and traumatic stress. Finally, some SLaM services provide to a wider geographic catchment (eg, Addiction services to Bexley and Greenwich boroughs) and others are catchment independent (eg, General Hospital Liaison services are provided to the four Acute Trusts within the catchment regardless of individual patients’ areas of residence).

Clinical records have been fully electronic (ie, paperless) across all SLaM services since April 2006, using the bespoke Patient Journey System (PJS) which incorporated legacy data from earlier service-specific EHRs. The CRIS application was developed in 2007–2008 and consists of a series of data-processing pipelines which both structure and de-identify PJS fields, rendering effectively anonymised data from the full clinical record available at the researcher interface, with search and database assembly functionality facilitated by a front end designed for non-technical use. The anonymisation process and its effectiveness, including the de-identification of open-text fields and the generation of a pseudonymised identifier (CRIS ID), have been described in detail.^8^ The wider patient-led oversight and security model have also been previously described and have not changed significantly since the SLaM BRC Case Register was set up.^7^ ^8^ Ethical approval as an anonymised database for secondary analysis was originally granted in 2008, and renewed for a further 5 years in 2013 (Oxford C Research Ethics Committee, reference 08/H0606/71+5). In terms of cohort coverage, all SLaM care is represented on CRIS. An opt-out model is in place for service users, and is advertised in all publicity material and initiatives; to date, only three people have requested this.

The SLaM BRC Case Register conforms to the WHO's formal description of a psychiatric case register—a ‘patient-centred longitudinal record of contacts with a defined set of psychiatric services originating from a defined population’,^9^ although its dynamic nature, updating against source files every 24 h, renders it distinct in some respects. The inclusion of both structured and unstructured (open-text) data in anonymised form, also variously distinguish the SLaM BRC Case Register from other local, regional and national case registries, including those extracted from EHRs such as the disease registries maintained by the US Veteran's Administration.^10^ ^11^ Routinely collected data resources such as the Mental Health Minimum Dataset and Hospital Episode Statistics (HES) for England and Wales overlap with SLaM Case Register data but are limited to prespecified structured fields.

### Early experience with CRIS and its influence on subsequent design

**Figure 1:**
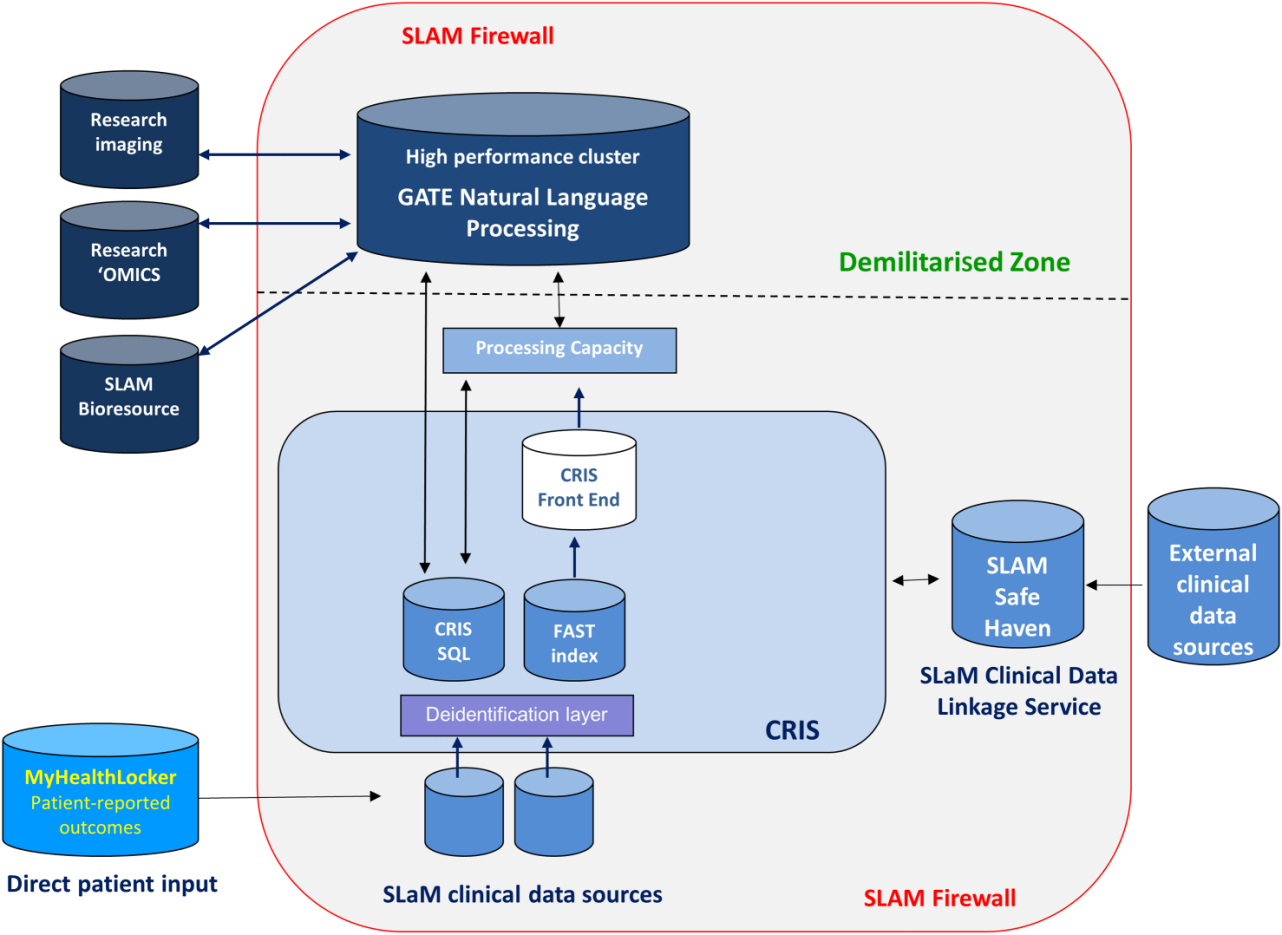
Diagram/map of CRIS technical architecture including natural language processing and data linkage. CRIS, Clinical Record Interactive Search; GATE, General Architecture for Text Engineering; SLaM, South London and Maudsley.

Developments in the technical architecture underlying CRIS are summarised in the online supplementary appendix and the current model is displayed in figure 1. Studies published to date using CRIS-derived data have generally fallen into two groups. The first have used a combination of open-text and structured data, with open-text data identified using search terms and then manually coded into numeric form for the purpose of analysis. Because of this, sample sizes have been limited to no more than several hundred. However, productive examples include one of the largest case series assembled of people with neuroleptic malignant syndrome, in order to evaluate the range of diagnostic criteria,^12^ and associations with antipsychotic exposure,^13^ as well as a study of factors associated with khat use in a comprehensive sample of Somali mental health service users.^14^ The second group of studies have used only structured data or have made very limited use of open-text data. These have typically analysed sample sizes of several thousand or more. Examples include studies of residential mobility and of homelessness among inpatients on mental health wards, and a series of investigations of mortality associated with mental disorder, described later.^15^ ^16^

10.1136/bmjopen-2015-008721.supp1 Supplementary appendix

Important experiential learning occurred during the initial stages of CRIS use. First, we found that it was sometimes desirable to select and combine data from records in ways that were unsupported by the original CRIS interface (eg, because of complex temporal relationships required between fields). Second, it became clear that while being able to identify and retrieve open-text records according to the presence of prespecified search terms did achieve helpful economy of effort, it did not remove the work needed to generate quantitative data from open text. Indeed, for those projects dependent on the use of open text, the manual coding process placed important limitations on sample size and study duration. Finally, researchers began to develop ideas that required data in addition to those stored in the source EHR, such as data from primary care, acute care and outcomes such as mortality. In the succeeding sections, we set out how the SLaM BRC Case Register has evolved to respond to these challenges.

### Handling open text

As outlined above, a priority for development has been to develop more efficient ways of using open-text data in the SLaM BRC Case Register. Early case register data collection included manually reading the de-identified text fields returned by CRIS, such as routine case notes, correspondence and medication notes. For example, one of the recent publications involved manually reading of 2860 records on CRIS of patients receiving acetylcholinesterase inhibitors in order to record their Mini-Mental State Examination (MMSE) scores and respective dates, and other medication prescribed.^17^ Through this process over 11 000 MMSE scores were ascertained; however, there were significant demands in terms of time and resources and the exercise was only possible as the focus of a PhD studentship. Beyond the efficiencies in manual coding gained by extracting only those records required for coding, through keyword searches and postsearch processing, further gains may be made by displaying text fields in ways that make text of interest easier to see, and by displaying data that are required to be reviewed together in close proximity, and away from other data. For example, in studies of homelessness and residential mobility among inpatients, 4485 admissions were selected according to defined criteria, and free-text records corresponding to these admissions were selected if they contained the terms ‘homeless’, ‘NFA’ or ‘no fixed abode’.^15^ ^16^ The aim was to check structured data on homelessness against free-text data, and if necessary, to supplement the former. SAS was used to insert ‘tags’ that change font colour (red) and weight (bold) for the target words when the data are displayed in Excel, allowing around 2000 free-text progress notes to be coded as homeless/not homeless in less than a day. A SAS Enterprise Guide project developed in collaboration with Amadeus Software Ltd allows CRIS users to do this via a graphical user interface.

A more ambitious approach has been followed for an ongoing project to capture incident cases of psychosis, supported by another Enterprise Guide project developed in collaboration with Amadeus Software Ltd. First, a structured query language (SQL) query retrieves a selection of data for individuals not already present on a cumulative database of first-episode psychotic patients and not already diagnosed as having a psychotic disorder, and whose recent free-text entries contain particular words of interest such as ‘delusion’ or ‘hallucination’. Second, these data are imported into SAS and then automatically outputted in a format suitable for manual coding. This involves splitting data into a multiworksheet Excel workbook, such that each worksheet (tab) contains only data relating to a single person (in the case of our proposed project, each worksheet would similarly pertain to a single episode of care). Targeted words are displayed in colour and in bold.

In contrast to the facilitated, but still manual approaches described above, natural language processing (NLP) techniques have been evaluated and applied for extracting knowledge from unstructured text data. For our purposes, the key NLP technique has been information extraction (IE) where unstructured text is converted into structured tables.^18^ Such methods promise massive reductions in the time resource required by researchers to unlock information held in clinical notes that in turn may be connected to other parts of the structured record. It was therefore decided, early in the postdevelopment phase, to implement a text-mining capability in CRIS. This was to be generic, in that information to be extracted could not necessarily be foreseen in advance of the design of individual research studies. General Architecture for Text Engineering (GATE) was chosen as the core NLP infrastructure for CRIS.^19^ ^20^ GATE is a widely used suite of open source software for text engineering that includes a workbench for developing applications, tools for distributing those applications on different computer hardware architectures, a quality assurance suite and facilities for manual preparation of example data.^19–21^ GATE’s origins are in clinical IE and it has been widely applied in this context.^22^ ^23^ GATE includes a flexible architecture for IE and text mining, a large set of pluggable text processing components, and graphical tools for organising those components into new applications. The GATE suite also includes tools for text-mining workflow, distributed processing and visualisation. A variety of text processing tools and document formats may be plugged into this architecture, with individual tools being chained together into processing ‘pipelines’, and documents processed in series through these pipelines.

Two distinct shallow language processing methodologies have been adopted for CRIS development, in collaboration with University of Sheffield Department of Computer Science. The first may be described as rule-based pattern matching of key concepts. Sentences are first processed to find and create annotations based on simple surface linguistic information (such as words, sentences, etc). This step is then followed by the process of finding concept-specific keywords, which are used to recognise likely sentences of importance to the IE task. For example, in an application to determine the smoking status of a patient implied by texts, such a dictionary, might list the terms of common tobacco products and activities—‘cigarette’, ‘smoker’, etc. Finally, a set of patterns specific to the text-mining task are run over the previously generated annotations in order to create a final annotation containing all of the information required in a readily extractable format. The challenge of the pattern matching approach is that it is knowledge intensive. A successful series of patterns need to be developed in relation to a specific IE task (eg, to extract medications, educational level or particular test results). They have to be built manually by GATE users with language engineering skills, using definitions agreed with clinicians and epidemiologists. A sample of the output from an initial prototype application is then corrected by a clinician or epidemiologist, which in turn is used to stimulate discussion about requirements and to provide a basis for multiple iterations of development until performance requirements are met. An advantage of this IE approach is that it also allows researchers to combine information available from open text and structured fields available in CRIS, through SQL, thus combining multiple sources of information. At the postprocessing stage, we can further apply specific filtering criteria to data extraction, such as frequency and length of prescribing and number of concomitant drugs, thus identifying more complex patterns in the text, such as antipsychotic medication profiles (ie, antipsychotic polypharmacy).^24^

Because of the lengthy development cycles of building shallow parsing algorithms, a second IE methodology has also been evaluated. Here, support vector machines (SVMs) are used to rapidly achieve respectable results for certain types of IE problem. A SVM is a machine-learning technique where the intention is to represent instances of text as vectors in high dimensional space. With a training set of instances labelled as indicative of a desired class, the SVM implementation in GATE generates a hyperplane which can in turn be used to classify unseen instances pertaining to the described class in the training set. In practice, this primarily uses a technique known as ‘bag of words’, where the occurrence of single words within a sentence is the principal currency used to distinguish the various classes. The first part of the model construction requires an expert (eg, clinician) to review a set of documents and label sentences which are relevant to the concept in question, in much the same way that they might signal to a language engineer the relevance of a given sentence for a pattern-based approach. The combination of labelled and unlabelled sentences forms the training data, from which the SVM learns the classification function. This model is then applied to unseen data, and the model quality assessed by human review. If required, further training data can be supplied, which may involve an active learning-inspired approach. A limitation with SVMs applied in CRIS has been that they have limited suitability for complex data extraction problems; however, in scenarios where the assertion to be extracted is simple and tend to be restricted to a concise set of clinical language, performance has been found to be very good and IE applications with immediate utility can be rapidly developed.^25^ The TextHunter program was designed specifically to aid the process of clinical text annotation in CRIS, providing an easy-to-use interface for annotators with a focus on the sentence containing the word(s) of interest and immediately proximal text and functionality for rapid coding into discrete groups, typically comprising the following: (1) positive (ie, implying that the construct is present); (2) negative (ie, a statement indicating that the construct is absent); and (3) irrelevant text.^26^ Additional TextHunter functionality includes platforms for interannotator agreement testing, and the creation of gold standard and test annotation sets.

Whether rules-based or machine-learning approaches are used, separate training and test data sets are constructed. Standard metrics for evaluating IE application performance in the test data sets, at the level of the individual text annotation, comprise **precision** (equivalent to positive predictive value; the proportion of IE application ‘hits’ which are found to identify the genuine construct) and **recall** (equivalent to sensitivity; the proportions of instances of the genuine construct which are identified by the application). Employing text mining within the CRIS data set has involved a trade-off between the two. However, the longitudinal nature of EHR data means that there are generally multiple opportunities for an NLP application to capture a piece of information; therefore, suboptimal recall can be compensated for and the focus has been on maximising precision. For the purpose of precision and recall testing, there are two reportable outcomes. The first is ‘annotation level’, which is carried out across randomly selected documents and is an indicator of the base level of performance of the application. This figure is useful for developmental purposes, or, in the case of simple concepts that do not require postprocessing, for estimating the final performance of the algorithm. The second type of precision and recall are ‘currency level’, measuring performance after postprocessing.

### The SLaM Clinical Data Linkage Service

SLaM comprises one part of the KHP AHSC (established with King's College London, Guy's and St Thomas’ and King's College Hospitals NHS Foundation Trusts) and received National Institute of Health Research (NIHR) funding to set up a service to meet the growing demand from SLaM and KHP researchers whose projects require linked data extracts. SLaM consequently established the Clinical Data Linkage Service (CDLS) as a trusted third party safe haven set up to enable safe and secure data processing services (linkage, and/or storage, and/or extraction) on distinct data sets for secondary research use. The two main methods of linkage have involved either (1) CDLS performing a secure linkage using deterministic or probabilistic matching if/as required or (2) CDLS supporting another trusted third party service to perform the linkage outside of the SLaM electronic firewall followed by CDLS receiving the linked data afterwards (eg, CRIS-HES linkage). Linked data are stored by CDLS in accordance with the SLaM ICT Security Policy and a set of standards contained in a CDLS Memorandum of Understanding completed by the data controllers providing data to individual projects, prior to undertaking any data processing for the project. Linked data are stored on a CDLS server within the SLaM firewall. To date, linkages have been successfully carried out between CRIS and a number of databases, described below.

#### Primary care (Lambeth DataNet)

Lambeth DataNet (LDN) has been used for several research studies.^27^ ^28^ Using the services of a contracted partner, Quality Medical Solutions (QMS) until April 2014, data are extracted and pseudonymised from the general practitioner (GP) practices in question. In terms of the mechanism of linkage, QMS scramble the patient identifiable information (NHS number) within the complete LDN data set and send the algorithm to the CDLS using an official encrypted NHS data transfer method to allow linked data files to be generated within CDLS. All identifying data other than CRIS and LDN pseudonyms are then removed. On final approval, SLaM BRC researchers will submit their data extract request to CDLS, either using CRIS to identify a discrete list of client pseudonyms for their project cohort to be linked with CRIS and LDN data (this pseudonym is not returned to the researcher), or submitting a detailed description of the cohort under investigation for CDLS to assemble the corresponding linked data. Once the linkage is complete, the LDN ID pseudonym is destroyed and an anonym (project-specific ID) is used thus creating a project-specific, fully anonymised data set for analysis. LDN currently extracts data from all GP practices in Lambeth—that is, around a quarter of the geographic catchment served by SLaM.

#### Department for Education National Pupil Database

The Education (Individual Pupil Information; Prescribed Persons; England) Regulations 2009 as amended by The Education (Individual Pupil Information; Prescribed Persons; England; Amendment) Regulations 2013 enable the Department for Education (DfE) to share individual pupil information from the National Pupil Database (NPD) with named bodies and persons who, for the purpose of promoting the education or well-being of children in England, are conducting research or analysis, producing statistics, or providing information, advice or guidance. Access is subject to requesters complying with terms and conditions imposed under contractual arrangements and a rigorous approvals process. The DfE Data Management Advisory Panel approved the DfE Data and Statistics division linkage service to undertake the linking of IDs between CRIS and the NPD. In terms of the data linkage mechanism, SLaM CDLS will first identify all children under 17 on the CRIS database, comprising approximately 35 000 cases who have attended SLaM Children and Adolescent Mental Health Services between 1 January 2008 and 31 December 2013. Identifiers will then be sent via secure file transfer to the DfE Data and Statistics Department who will match these against the NPD identifiers cohort (approximately 15 million records), generating a pupil-specific, non-identifiable NPD ID variable across the whole data set, and adding the CRIS ID to this table for cases only, stripping the resultant table of all identifiers other than the anonymised NPD ID and the pseudonymised CRIS ID, and transferring the data set back to SLaM CDLS using secure file transfer. Researchers on approved projects will compile clinical data from CRIS for approved analyses and send to CDLS for linking. CDLS will then fully anonymise resultant tables by replacing the CRIS ID for cases throughout with a project-specific CDLS ID, and the link between the CRIS ID and CDLS ID will be permanently destroyed prior to sending linked tables to researchers for analysis.

#### Hospital Episode Statistics

HES data are compiled from all NHS Trusts in England (both acute and mental health services), including statistical abstracts of records of all inpatient episodes, as well as outpatient and emergency care. For this linkage, CRIS identifiers are compiled by CDLS, and transferred to the Health and Social Care Information Centre (HSCIC) using an NHS-approved secure file transfer protocol. HSCIC then adds the CRIS ID to all HES records that match CRIS records and extracts all other HES records for patients within the four catchment boroughs served by SLaM (the control group). HSCIC destroys patient identifiers leaving only the CRIS ID and HES extract ID. As with other linked data sets, the CRIS-HES data are transferred back to CDLS to be held and provided to researchers in a fully anonymised format.

#### Mortality

Office for National Statistics (ONS) mortality data are additionally requested via the HSCIC. CDLS send identifiers (CRIS ID, first name, last name, date of birth, gender, postcode and NHS number) to HSCIC, who return ONS mortality data to CDLS via the same secure file transfer protocol as that used for the HES linkage. While ONS mortality data include details of information recorded on the death certificate, date of death is available on a wider CRIS sample through data held by SLaM, in common with most mental health NHS Trusts through standard linkage of all NHS numbers to the national spine.

#### Cancer

In an initial piece of work, a data linkage was set up between CRIS and Thames Cancer Register by the UK Government Department of Health Research Capability Programme, findings from which have been previously reported and which generated an irreversibly anonymised linked data set.^29^ This data resource is currently being expanded to bring together updated local data from the National Cancer Registration Service (NCRS) held by Public Health England's London Knowledge and Intelligence Team, linking this with CRIS and incorporating additional HES and mortality data provided by HSCIC and ONS.

#### Procedures and resources

Results from all these linkages are stored within the CDLS safe haven, and CDLS plays a key role in wider governance, supplementing the role of CRIS-specific oversight and data security previously described.^7^ ^8^ While set up to support research at the SLaM BRC, as an independent trusted third party service CDLS sits outside the BRC and is managed by a dedicated team within the SLaM Information and Communications Technology department, reporting directly to the SLaM Director of ICT Strategy and ultimately accountable to the SLaM Trust Board. Important features of CDLS work are the secure handling and storage of identifier fields required for data linkage. Section 251 (s.251) of the NHS Act 2006 allows the common law duty of confidentiality to be set aside in specific circumstances where anonymised information is not sufficient and where patient consent is not practicable. S.251 approval has been granted to SLaM for all the above linkages, which allow data to be available in an identifiable format to a small number of data processing staff in accordance with data sharing contracts. Activity for projects using linked data sets held by CDLS is audited by the CDLS Safe Haven Officer, helping to ensure that the user's project requirements (eg, clinical research, surveillance, service improvement or audit) are met, and projects progress within the agreed policy and practice framework. The CDLS communications plan has a patient-facing aspect in raising awareness of the projects facilitated by the CDLS. Service user involvement is ensured in the decision-making process of approving projects working with linked data held by CDLS, and the patient-chaired CRIS Oversight Committee reviews and approves all projects using CRIS-linked data. Separate committees with the same terms of reference have been set up to provide governance for the LDN and NPD linkages, in order to accommodate representation from respective agencies providing these data.

Four distinct services are thus offered by the CDLS. First, CDLS provides advice on permissions, approvals and contracts. These include consideration of academic, technical, legal and ethical requirements. The SLaM ‘Caldicott Guardian’ is responsible for any use of patient identifiable information and their approval is also a prerequisite. Second, CDLS facilitates data linkages either within the CDLS safe haven or via a third party, coordinating the secure transfer of data. Third, CDLS is responsible for the secure storage of linked data in accordance with predefined information governance and security standards. Fourth, CDLS as the custodian for the linked data prepares and extracts bespoke and prespecified databases for approved CRIS projects and provides these to researchers. Therefore, there is no direct access by researchers to the full linked data files, enhancing data protection and confidentiality.

### Cohort characteristics

Initial descriptive data were assembled on the catchment area for SLaM (Croydon, Lambeth, Lewisham and Southwark) using publicly available sociodemographic information from ONS census data.^30^ Analyses of CRIS data used 31 December 2014 as a census date for descriptive statistics including sociodemographic and diagnostic profiles. ‘Active’ patients on this date were defined as those who had been referred to and accepted by SLaM and had not been discharged by 31 December 2014. ‘Inactive’ patients had a recorded activity date on or before 31 December 2014 and excluded referrals categorised as ‘rejected’ or ‘waiting’. On 31 December 2014, 223 224 patient records were available on CRIS, of which 31 961 described ‘active’ patients and 191 263 ‘inactive’. The remaining 21 882 records described referrals, which were either solely characterised as ‘rejected’ or ‘waiting’, and in which no team episode (for outpatients) or ward stay (for inpatients) was indicated. Descriptive data were further provided for key linked data sets at that time. In this respect, the most recent mortality date recorded in the linked ONS mortality data set was 16 December 2013; cancer registry data were linked up to 31 December 2008; HES data were available to 31 March 2013. For analyses of linked HES data, contacts with mental health services were excluded.

Descriptive data from the UK Census for the catchment populations served by SLaM are summarised in table 1 and contextualised with the same information for London as a whole and for England. There are slight differences in population structure between the four boroughs served, with Croydon having higher proportions of young children and older residents compared with London and the other three boroughs. Highest proportions in the young adult (20–39 year) age range were living in Lambeth and Southwark. As a whole, the SLaM catchment has a slightly higher predominance of working adults in the 20–59-year range compared with London, and shares with London lower proportions in older age ranges compared with England. The SLaM catchment has substantially higher proportions of residents from minority ethnic groups and/or born outside UK compared with England, whereas compared with London as a whole, there are higher proportions from black minority groups and lower proportions from Asian groups. In common with London as a whole, proportions are higher in both highest and lowest socioeconomic groups compared with England; proportions in unemployment are higher, but so are proportions with higher levels of education. Of the catchment boroughs, Lambeth, Southwark and Lewisham have higher levels of both in-migration and out-migration compared with Croydon. Based on the ratios between summed borough statistics and those for the catchment overall, 76.9% of inflow migration and 78.5% of outflow migration was from/to areas outside the catchment, rather than between catchment boroughs.

**Table 1:** Descriptive statistics, derived from the 2011 UK Census, for the four London boroughs served by SLaM, compared with statistics for London and England as a whole

	SLaM catchment					Comparison statistics	
	Lambeth	Croydon	Lewisham	Southwark	Combined	London	England
Total population*	310 200	368 900	281 600	293 500	1 254 200	8 308 400	53 493 700
Age (%)							
<20	21.7	26.9	25.4	23.0	24.4	24.5	24.0
20–39	44.2	29.3	36.3	41.7	37.5	35.8	27.0
40–59	23.4	26.9	25.3	24.4	25.1	24.5	26.7
60–79	8.6	13.5	10.3	8.8	10.4	12.1	17.7
≥80	2.1	3.4	2.7	2.1	2.6	3.1	4.6
Gender (%)							
Male	49.8	48.5	48.9	49.5	49.1	49.3	49.2
Female	50.2	51.5	51.1	50.5	50.9	50.7	50.8
Education† (%)							
No qualifications	14.2	17.6	17.7	16.3	16.5	17.6	22.5
Highest level of qualification; level 1 qualifications	8.5	13.8	11.1	9.4	10.9	10.7	13.3
Highest level of qualification; level 2 qualifications	9.8	15.2	12.5	10.2	12.1	11.8	15.2
Highest level of qualification; apprenticeship	1.1	2.1	1.4	1.2	1.5	1.6	3.5
Highest level of qualification; level 3 qualifications	9.7	11.4	10.8	10.5	10.6	10.5	12.4
Highest level of qualification; level 4 qualifications and above	46.6	31.8	38	43.1	39.5	37.7	27.4
Highest level of qualification; other qualifications	10.1	8.1	8.5	9.3	9.0	10.1	5.7
Self-assigned ethnicity (%)							
White	57.1	55.2	53.5	54.3	55.1	59.8	85.5
Mixed	7.6	6.4	7.4	6.2	6.9	5.1	2.2
Asian or Asian British	6.8	16.4	9.3	9.5	10.8	18.4	7.7
Black or Black British	25.9	20.2	27.2	26.8	24.7	13.3	3.4
Other	2.6	1.8	2.6	3.2	2.5	3.4	1.2
Socioeconomic classification (%)‡							
Higher managerial, administrative and professional occupations	16.2	14.1	13.1	15.8	14.8	15.8	13.8
Lower managerial, administrative and professional occupations	27.3	24.8	25.7	24.8	25.6	24.7	22.8
Intermediate occupations	10.6	13.7	12.1	10.3	11.8	10.9	10.5
Small employers and own account workers	9.7	12.9	10.9	8.8	10.7	12.9	12.8
Lower supervisory and technical occupations	5.9	6.7	6.8	6.6	6.5	6.5	8.8
Semiroutine occupations	10.3	12	12.6	12	11.7	10.9	13
Routine occupations	9.7	8.3	8.7	9.9	9.1	8.8	12.1
Never worked and long-term unemployed	6.9	5.3	6.4	7	6.3	6.5	4.2
Full-time students	3.4	2.2	3.7	4.8	3.4	3	2
Percentage of people born in UK	61.1	70.4	66.4	63.2	65.5	85.8	94.1
Estimated migration (thousands per year) for the 1 year period ending June 2014§							
Inflow	29.07	19.19	21.2	25.25	72.81	196.6	526
Outflow	31.78	19.81	22.36	27.53	79.71	251.6	314
Balance	−2.71	−0.62	−1.16	−2.28	−6.90	−55	+212

*Resident population estimates by broad age band, mid-2013, using ONS 2011 census.

†All usual residents aged over 16 on the census date 27 March 2011.

‡Based on HRP: an individual person within a household to act as a reference point and charactering whole household according to characteristics of the chosen reference person.

§Data source: http://www.ons.gov.uk/ons/publications/re-reference-tables.html?edition=tcm%3A77-326817 accessed on the 5 November 2015. SLaM catchment and London statistics calculated for the 1 year period ending June 2013 (and the overall catchment statistic does not include within-catchment migration); England figures represent rolling annual data for year ending June 2014.

HRP, household reference person; ONS, Office for National Statistics; SLaM, South London and Maudsley.

**Figure 2:**
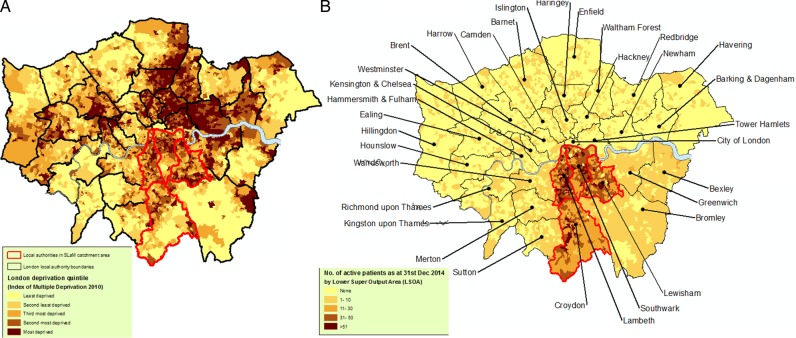
Maps contextualising deprivation levels in the South London and Maudsley (SLaM) catchment compared with London as a whole, and illustrating the distribution of recorded residences for active patients (on 31 December 2014) within London.

**Figure 3:**
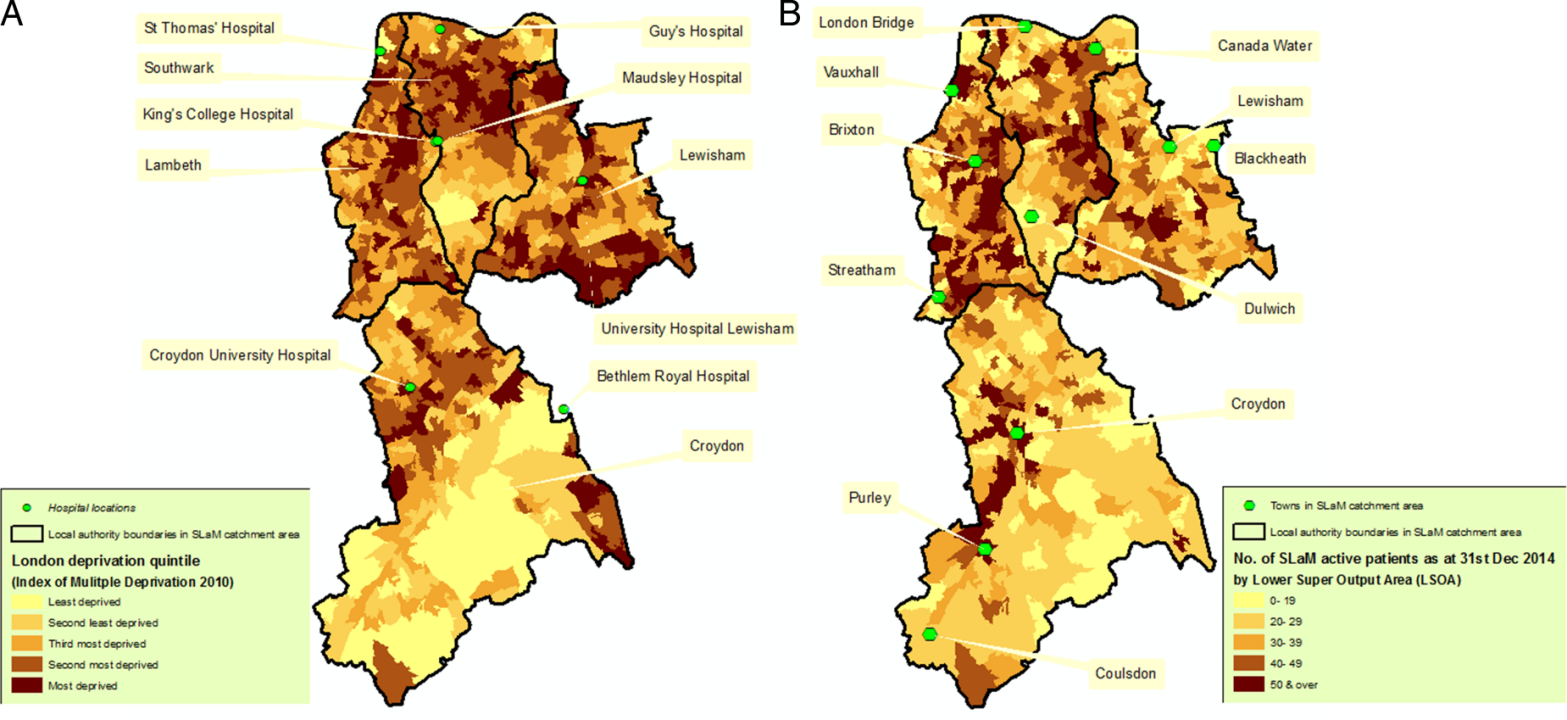
(A, B) Maps showing distribution of deprivation levels in the four catchment boroughs served by South London and Maudsley (SLaM), the key hospital sites and the number of active patients (on 31 December 2014) across the same geography.

**Figure 4:**
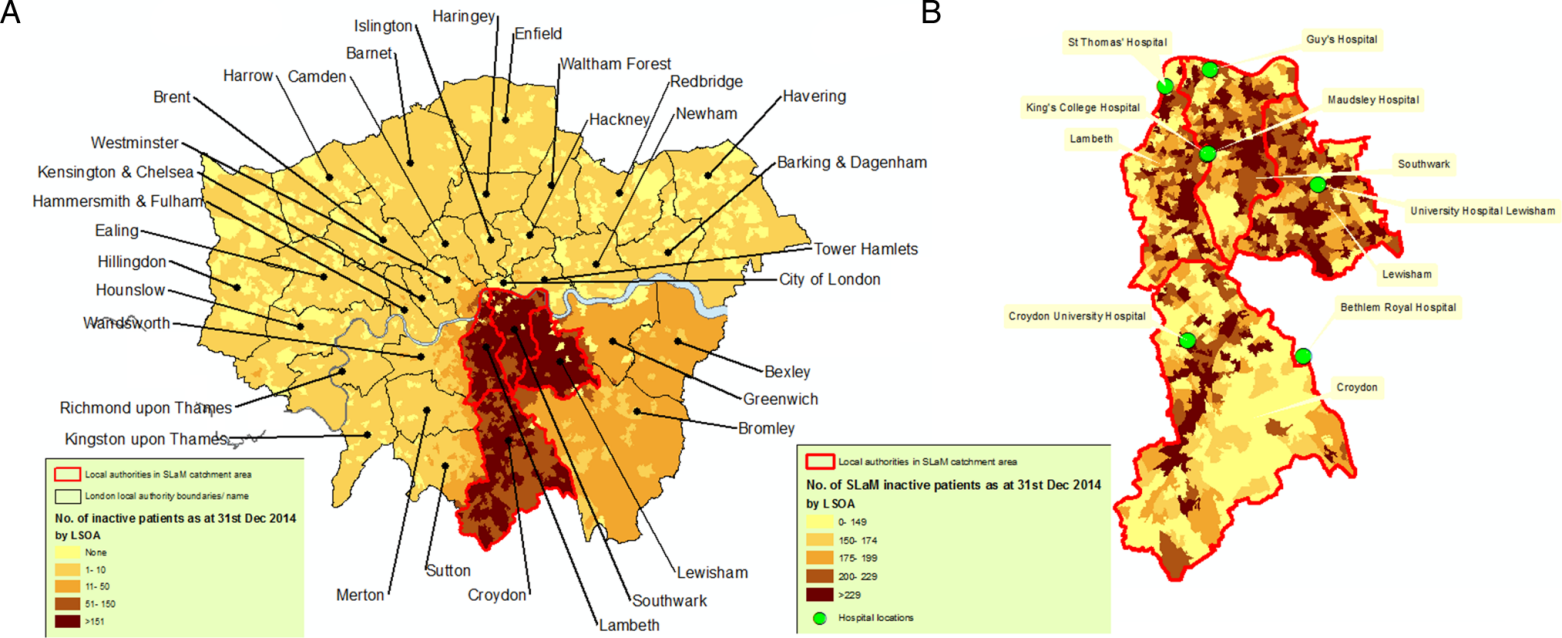
Maps illustrating the distribution of recorded residences for inactive patients (on 31 December 2014) within London and SLaM catchment area. LSOA, lower super output area; SLaM, South London and Maudsley.

Geographic characteristics are summarised in figures 2–4. Figure 2A visually contextualises deprivation levels in SLaM compared with other areas of London, and figure 2B summarises the most recently recorded residence of active SLaM patients. In the latter, most active SLaM patients were identified as residing within its geographic catchment, although appreciable numbers were drawn from a wider geography. Within the SLaM catchment, higher numbers of active patients were generally found in areas of higher deprivation, although several anomalous areas can be seen—for example, those with high deprivation and relatively low numbers of active patients (figure 3A, B). Figure 4 illustrates the most recent recorded residence of non-active patients in London (figure 4A) and specifically in SLaM's catchment (figure 4B). Outside SLaM's catchment, relatively high numbers of inactive patients were recorded as residing in neighbouring local authorities in South East London including Bexley, Greenwich and Bromley.

Descriptive data are summarised in table 2 for all people who were represented on the SLaM BRC Case Register on 31 December 2014. Higher proportions of active patients were 80 years and older and in the 40–59 group compared with proportions in the four catchments. Compared with the catchment area characteristics described in table 1, active SLaM patients had a slightly higher male predominance, and there were higher proportions self-assigning as white, mixed or other ethnicity. Around 70% were single. Employment status data were available on less than 25% of the active sample, but of this group around 66% were unemployed. Of active SLaM patients on the census date, 6574 (20.9%) were either residing in boroughs outside London or living in London but outside SLaM's four catchment boroughs. Of these, 3385 (51.5%) were in contact with SLaM services that provided for other boroughs, 1941 (29.5%) were using one or more of SLaM's national services, 341 (5.2%) were in contact with General Hospital Liaison services covering one of the four Acute Trusts within the SLaM catchment, and 907 (13.8%) were previous catchment residents currently living outside the catchment (193 of whose addresses were recorded as temporary).

**Table 2:** Characteristics of patients represented on the South London and Maudsley National Health Service (NHS) Foundation Trust Biomedical Research Centre (SLaM BRC) Case Register records (census date: 31 December 2014)

	Active patients (%)	Inactive patients* (%)
Characteristic	N=31 961	N=191 263
Current age (years)		
<20	6265 (19.6)	23 740 (12.4)
20–39	9464 (29.6)	65 493 (34.2)
40–59	10 101 (31.6)	59 336 (31.0)
60–79	4017 (12.6)	23 924 (12.5)
≥80	2114 (6.6)	18 770 (9.8)
Year of birth		
On or after 1994	6785 (21.2)	27 214 (14.2)
1993–1973	10 032 (31.4)	68 722 (35.9)
1973–1954	9337 (29.2)	53 317 (27.9)
1953–1934	3913 (12.2)	22 065 (11.5)
On or after 1933	1894 (5.9)	19 945 (10.4)
Gender		
Male	16 780 (52.5)	93 902 (49.1)
Female	15 160 (47.5)	97 327 (50.9)
Self-assigned ethnicity (full breakdown)†		
British	14 833 (50.5)	83 425 (55.6)
Irish	614 (2.1)	3819 (2.5)
Any other white background	2196 (7.5)	13 072 (8.7)
Mixed: white and black	770 (2.6)	2899 (1.9)
Mixed: white and Asian	104 (0.4)	421 (0.3)
Mixed: any other mixed background	277 (0.9)	961 (0.6)
Indian	413 (1.4)	2072 (1.4)
Pakistani	211 (0.7)	958 (0.6)
Bangladeshi	115 (0.4)	631 (0.4)
Any other Asian background	596 (2.0)	3105 (2.1)
Caribbean	2192 (7.5)	7654 (5.1)
African	2156 (7.3)	9178 (6.1)
Any other black background	2923 (10)	10 628 (7.1)
Chinese	107 (0.4)	593 (0.4)
Any other ethnic group	1865 (6.3)	10 715 (7.1)
Ethnicity not known or not stated	2589 (8.8)	41 132 (21.5)
Self-assigned ethnicity (amalgamated)†		
British, Irish or any other white ethnic groups	17 643 (60.1)	100 316 (52.4)
Mixed	1151 (3.9)	4281 (2.2)
Indian, Pakistani, Bangladeshi or ‘other Asian’	1335 (4.5)	6766 (3.5)
Caribbean, African or any ‘other black’	7271 (24.8)	27 460 (14.4)
Other	1972 (6.7)	11 308 (5.9)
Area of most recently recorded residence‡		
Croydon	6127 (19.5)	36 996 (20.4)
Lambeth	7043 (22.4)	33 471 (18.5)
Lewisham	5610 (17.8)	35 206 (19.4)
Southwark	6120 (19.4)	32 961 (18.2)
Other London boroughs	3179 (10.1)	27 012 (14.9)
Outside London	3395 (10.8)	15 649 (8.6)
Unknown	487 (1.5)	9968 (5.2)
Most recent employment status		
Paid employment	439 (6)	5118 (13.4)
Part-time employment	114 (1.6)	581 (1.5)
Self-employed	31 (0.4)	408 (1.1)
Volunteer	67 (0.9)	95 (0.2)
Government training scheme	<10 (0.1)	24 (0.1)
Full-time student	204 (2.8)	1623 (4.2)
Full-time student—school age	930 (12.7)	7725 (20.2)
Retired	504 (6.9)	6790 (17.8)
Registered disabled	71 (1.0)	352 (0.9)
Unemployed	4827 (66.1)	14 949 (39.1)
Other	115 (1.6)	534 (1.4)
Employment status not known	24 654 (77.1)	153 064 (80.0)
Most recent marital status		
Married	1329 (4.7)	13 701 (10.4)
Married/civil partner	3111 (11)	11 027 (8.3)
Cohabiting	556 (2.0)	2532 (1.9)
Divorced	622 (2.2)	3920 (3.0)
Divorced/civil partnership dissolved	633 (2.2)	2293 (1.7)
Separated	853 (3.0)	5303 (4.0)
Widowed	320 (1.1)	5985 (4.5)
Widowed/surviving civil partner	1046 (3.7)	4280 (3.2)
Single	19 763 (70)	83 319 (62.9)
Marital status not known or not disclosed	3728 (11.7)	58 903 (30.8)

*Inactive: those not currently receiving treatment and who have been discharged from all services.

†Excluding those not stated or none: active=2589/31961, inactive=41 132.

‡As at 31 December 2014.

On the 31 December 2014 census date, there were nearly 32 000 active cases receiving care from SLaM services, with the largest numbers receiving care from Psychosis or Child and Adolescent Mental Health Services (table 3). A further 190 000 plus patients on the SLaM BRC Case Register were inactive to SLaM, nearly one-third of whom received care from Psychological Medicine services (which includes General Hospital Liaison services). Table 4 provides an additional description of overlap between services for active and inactive patients, with over 1000 active patients in contact with two or more specialties concurrently and over 15 000 inactive patients having received care from two or more specialties. Ever-recorded primary diagnoses are summarised in table 5. Of active patients, the most common mental disorder diagnoses ever recorded were schizophrenia (21.2%) and mood (19.0%) disorders, followed by organic (11.0%), substance use (11.7%) and neurotic (13.0%) disorders, and disorders of childhood and adolescence (11.3%). Sizes of data linkage samples are described in tables 6–8. Nearly 85% of CRIS patients had records in HES (excluding mental health service data) and nearly 2% of CRIS patients had data linked to those from the cancer registry within the years of data availability (table 6). Distributions of underlying cause of death are summarised in table 9 for the linked sample with this information, and primary cancer diagnoses are similarly described in table 10.

**Table 3:** Characteristics of active and inactive cases on the South London and Maudsley National Health Service (NHS) Foundation Trust Biomedical Research Centre (SLaM BRC) Case Register: most recent specialty (census date: 31 December 2014)*

Current or most recent SLaM specialty service providing care	Number (%)	
	Active patients	Inactive patients†
Psychosis	7116 (22.3)	12 444 (6.5)
Child and Adolescent Mental Health Services	5765 (18.0)	27 231 (14.2)
Mood, Anxiety and Personality	5271 (16.5)	31 887 (16.7)
Mental Health of Older Adults and Dementia	4217 (13.2)	24 842 (13.0)
Psychological Medicine	4333 (13.6)	59 212 (31.0)
Addictions	2559 (8.0)	12 768 (6.7)
Behavioural and Developmental Psychiatry	3532 (11.1)	7898 (4.1)
Unknown/not recorded	719 (2.2)	80 440 (42.1)

*Some patients may have records with more than one specialty.

†Inactive: those not currently receiving treatment and who have been discharged from all services.

**Table 4:** Characteristics of active and inactive cases on the South London and Maudsley National Health Service (NHS) Foundation Trust Biomedical Research Centre (SLaM BRC) Case Register: patterns of multispecialty care (census date: 31 December 2014)

	Number of specialties involved (current or most recent status)							
	Active patients			Inactive patients				
Specialty	1	2*	3+*	1	2*	3*	4*	5+*
Addictions	2349	197	13	9348	2181	903	315	21
Behavioural and Developmental Psychiatry	3347	178	<10	6484	962	324	114	14
Child and Adolescent Mental Health Services	5671	86	<10	24 299	2275	521	128	<10
Mental Health of Older Adults and Dementia	4173	42	<10	22 360	2159	261	55	<10
Mood, Anxiety and Personality	4653	582	36	19 595	8670	3105	493	24
Psychological Medicine	3818	481	34	40 778	14 199	3690	521	24
Psychosis	6509	580	27	4644	4522	2773	482	23
Total	30 520	1073	41	127 508	17 484	3859	527	24

*Include multiple counts of patients.

**Table 5:** Characteristics of active and inactive cases on the SLaM BRC Case Register: primary diagnoses ever recorded (census date: 31 December 2014)*

	Number (%)	
Assigned primary diagnosis (ICD-10 code and description)	Active patients	Inactive patients†
F0–F09—organic, including symptomatic, mental disorders	3517 (11.0)	19 535 (10.2)
F10–F19—mental and behavioural disorders due to psychoactive substance use	3742 (11.7)	19 204 (10.0)
F20–F29—schizophrenia, schizotypal and delusional disorders	6778 (21.2)	10 069 (5.3)
F30–F39—mood (affective) disorders	6076 (19.0)	31 119 (16.3)
F40–F48—neurotic, stress-related and somatoform disorders	4155 (13.0)	22 800 (11.9)
F50–F59—behavioural syndromes associated with physiological disturbances and physical factors	1025 (3.2)	5800 (3.0)
F60–F69—disorders of adult personality and behaviour	1518 (4.7)	4078 (2.1)
F70–F79—mental retardation	807 (2.5)	2050 (1.1)
F80–F89—disorders of psychological development	1483 (4.6)	4405 (2.3)
F90–F98—behavioural and emotional disorders with onset usually occurring in childhood and adolescence	3607 (11.3)	10 343 (5.4)
Unspecified mental disorder	7016 (22.0)	28 122 (14.7)
No axis 1 diagnosis	526 (1.6)	6399 (3.3)
G—diseases of the nervous system	173 (0.5)	543 (0.3)
Other illness codes (A–E, H–Q)	669 (2.1)	7292 (3.8)
Symptoms, signs and abnormal clinical and laboratory findings, not elsewhere classified	101 (0.3)	1164 (0.6)
S–Y—injury, poisoning and external causes	398 (1.2)	1416 (0.7)
Z—factors influencing health status and contact with health services	6384 (20.0)	42 552 (22.2)
Number of patients with a primary diagnosis recorded (% of all patients)	29 820 (93.3)	157 027 (82.1)

*Some patients may have had more than one primary diagnosis recorded.

†Inactive: those not currently receiving treatment and who have been discharged from all services.

ICD, International Classification of Diseases; SLaM BRC, South London and Maudsley National Health Service (NHS) Foundation Trust Biomedical Research Centre.

**Table 6:** Number of patients represented on the SLaM BRC Case Register with CRIS data linked to other data sets

Data linkage	Number of patients on both databases (% of all CRIS active and inactive patients)
CRIS* and ONS mortality† data	20 864 (9.3)
CRIS* and HES‡ data	188 447 (84.4)
CRIS* and cancer registry§ data	3442 (1.5)

*CRIS active and inactive patients recorded as at 31 December 2014.

†(Up to 16 of December 2013.) ‡Up to 31 March 2013. §(Cancer registry data last updated 31 December 2008).

CRIS, Clinical Record Interactive Search; HES, Health Episode Statistics; ONS, Office for National Statistics; SLaM BRC, South London and Maudsley National Health Service (NHS) Foundation Trust Biomedical Research Centre.

**Table 7:** Number of people represented on the SLaM BRC Case Register with linked HES data

	CRIS data*	
HES data†	Active (%)	Inactive (%)
Any inpatient care‡	18 387 (57.5)	137 577 (71.9)
Any emergency room attendance‡	18 139 (56.8)	129 041 (67.5)
Any outpatient attendance‡	20 642 (64.6)	150 748 (78.8)

*CRIS active and inactive patients recorded as at 31 December 2014.

†Excluding mental health inpatient/outpatient services.

‡Excluding mental health providers.

CRIS, Clinical Record Interactive Search; HES, Health Episode Statistics; SLaM BRC, South London and Maudsley National Health Service (NHS) Foundation Trust Biomedical Research Centre.

**Table 8:** Number of people represented on the SLaM BRC Case Register with linked HES and mortality data

Data linkage sample	Number of deaths (%)	
	Total	Linked to ONS mortality records*
People in CRIS† with at least one inpatient admission in HES	20 541 (9.2)	19 910 (8.9)
People in CRIS† with at least one A&E attendance record in HES	14 791 (6.6)	14 279 (6.4)
People in CRIS† with at least one outpatient record in HES	19 220 (8.6)	18 613 (8.3)

*Up to 16 of December 2013.

†All CRIS active and inactive patient deaths recorded up to 16 December 2013.

A&E, accident and emergency; CRIS, Clinical Record Interactive Search; HES, Health Episode Statistics; ONS, Office for National Statistics; SLaM BRC, South London and Maudsley National Health Service (NHS) Foundation Trust Biomedical Research Centre.

**Table 9:** Number of deaths in SLaM linked with ONS mortality data by underlying primary cause of death (latest date of record is as at 16 of December 2013)

ICD-10 chapter description (underlying cause of death)	Number of patients (% of all deaths in CRIS) (N=20 864)
Benign neoplasms or diseases of the blood	159 (0.8)
Cancers	3356 (16.1)
Certain conditions originating in the perinatal period and pregnancy, childbirth and the puerperium	<10
Codes for special purposes (eg, antibiotic resistance)	45 (0.2)
Congenital malformations, deformations and chromosomal abnormalities	73 (0.3)
Diseases of the circulatory system	5665 (27.2)
Diseases of the digestive system	1467 (7)
Diseases of the genitourinary system	689 (3.3)
Diseases of the musculoskeletal system	241 (1.2)
Diseases of the nervous system	1338 (6.4)
Diseases of the respiratory system	2964 (14.2)
Diseases of the skin	80 (0.4)
Endocrine, nutritional and metabolic diseases	445 (2.1)
External causes	1294 (6.2)
Infectious and parasitic diseases	356 (1.7)
Mental and behavioural disorders	2206 (10.6)
Symptoms and sign not elsewhere classified	376 (1.8)
Unknown/ missing	102 (0.5)

CRIS, Clinical Record Interactive Search; ICD, International Classification of Diseases; ONS, Office for National Statistics; SLaM, South London and Maudsley.

**Table 10:** Numbers of patients with both CRIS and cancer registry data, by primary cancer diagnosis (linkage last updated 31 December 2008)

Primary diagnosis (ICD-10 3-digit description)	Number (%) of patients
Malignant neoplasm of breast	563 (16.4)
Carcinoma in situ of cervix uteri	394 (11.4)
Malignant neoplasm of prostate	391 (11.4)
Malignant neoplasm of bronchus and lung	306 (8.9)
Malignant neoplasm of colon	179 (5.2)
Other malignant neoplasms of skin	152 (4.4)
Malignant neoplasm of bladder	92 (2.7)
Malignant neoplasm of rectum	90 (2.6)
Malignant neoplasm of corpus uteri	71 (2.1)
Malignant neoplasm of kidney, except renal pelvis	70 (2.0)
Other and unspecified types of non-Hodgkin’s lymphoma	65 (1.9)
Malignant melanoma of skin	58 (1.7)
Malignant neoplasm of brain	57 (1.7)
Malignant neoplasm of pancreas	53 (1.5)
Malignant neoplasm of stomach	53 (1.5)
Malignant neoplasm without specification of site	53 (1.5)
Malignant neoplasm of oesophagus	50 (1.5)
Malignant neoplasm of cervix uteri	48 (1.4)
Malignant neoplasm of ovary	46 (1.3)
Diffuse non-Hodgkin’s lymphoma	42 (1.2)
Myeloid leukaemia	42 (1.2)
Lymphoid leukaemia	39 (1.1)
Multiple myeloma and malignant plasma cell neoplasms	36 (1.0)
Malignant neoplasm of larynx	34 (1.0)
Other diagnoses	458 (13.3)

CRIS, Clinical Record Interactive Search; ICD, International Classification of Diseases.

### Performance of NLP applications

Performances of IE applications to date are summarised for CRIS as a whole, supplementary to more detailed publications on some of these.^31^ ^32^ ^33^ ^34^ The first NLP IE application to be developed was for the MMSE, a commonly used 0–30-point assessment of global cognitive function. The objective of the application was to ascertain both the numerator and denominator scores (because denominator scores of less than 30 are used where some items cannot be attempted because of, eg, sensory impairment), as well as the date implied for the assessment (because clinical text fields commonly refer to previous as well as current scores). Further rules for application postprocessing were that only MMSE scores with denominators over 25 were included (because scores below that level imply substantial missing data and a scale that was probably incompletely administered), and scores were excluded if two different numerators were assigned to the same date.^34^ The application for educational attainment sought to ascertain the numeric value associated with text commenting on school leaving age, whether the age itself or the year, and the application for ‘living alone’ simply sought to identify that phrase or equivalents applied to the patient. In developing the smoking application, authors extracted information from open-text fields, classifying patients as either ‘currently smoking’, ‘past smoker’ or ‘has never smoked’, with smoking of substances other than tobacco (eg, marijuana/cannabis and cocaine) specifically excluded.^31^ The methodology used an iterative process of manual ‘gold standard’ annotation of free-text documents, followed by comparison with the results generated by the application at each development stage, with analysis of this comparison feeding further development of the rules. The application for ‘diagnosis’ sought simply to extract any text strings associated with a diagnosis statement in order to supplement the existing structured (International Classification of Diseases (ICD)-10) fields. Its performance was evaluated formally in a random sample of 75 documents for ‘vascular dementia’,^33^ but is recommended for individual further evaluation in other conditions. The application for ascertaining pharmacotherapy was developed using a gazetteer of generic and commercial names for all medications in UK use in order to ascertain instances where the patient was reported as receiving these, with supplementary rules for ascertaining recorded dose, frequency/timing and starting/stopping statements. Its precision was first tested for clozapine receipt against a manual search of 279 documents, and recall was ascertained on a random set of 200 documents containing the word clozapine and scrutinised to ascertain an actual prescription.^32^ Finally, the validity of this application was recently further evaluated for six antipsychotic agents (amisulpiride, flupentixol, haloperidol, olanzapine, risperidone, zuclopenthixol) on instance level (ie, specific mentions in the text at individual points in time). To estimate precision and recall, the authors examined a subset of 20 patients for each medication, totalling 120 patients (the instances of antipsychotic prescribing varied from 328 to 1150 instances by antipsychotic agent) by running the NLP application over the set of unseen documents and comparing the results to the manual coding of the same data set.^24^ For all evaluations, an F-statistic was additionally calculated, representing the harmonic mean of precision and recall, and defined as: F=2×(precision×recall/(precision+recall)). As with the diagnosis application, further bespoke validation of the pharmacotherapy application is recommended for new medications or classes. Performance data are summarised for NLP IE applications in table 11, and table 12 describes the resulting additional structured data points generated across CRIS using these applications.

**Table 11:** Performance of natural language processing information extraction applications developed to date in the SLaM BRC Case Register

Application name	Construct sought	Number of patients tested	Precision	Recall	F-statistic
Smoking^31^	Is the patient a current smoker?	100	0.93	0.58	0.72
Clozapine—current use^32^	Is the patient currently using clozapine (within 3 months)?	Precision: 279, recall: 200	0.96	0.92	0.94
Clozapine—ever used^32^	Has the patient used clozapine in the past?	Precision: 279, recall: 200	0.99	0.92	0.95
Diagnosis^33^	What text accompanies a statement about diagnosis?	75	0.99	0.98	0.99
MMSE^34^	What MMSE score did the patient attain on a given date?	100	0.97	0.98	0.97
Education	What age did a patient leave school?	Precision: 100, recall: 115	0.95	0.59	0.73
Living alone	Is the patient living alone?	100	0.93	0.99	0.96
Amisulpride^24^	Is the patient currently using amisulpride?	20 patients with 619 instances	0.97	0.61	0.75
Flupentixol^24^	Is the patient currently using flupentixol?	20 patients with 328 instances	0.94	0.77	0.85
Haloperidol^24^	Is the patient currently using haloperidol?	20 patients with 747 instances	0.94	0.57	0.71
Olanzapine^24^	Is the patient currently using olanzapine?	20 patients with 1150 instances	0.95	0.69	0.80
Risperidone^24^	Is the patient currently using risperidone?	20 patients with 737 instances	0.95	0.64	0.76
Zuclopenthixol^24^	Is the patient currently using zuclopenthixol?	20 patients with 390 instances	0.97	0.68	0.80

MMSE, Mini-Mental State Examination; SLaM BRC, South London and Maudsley National Health Service (NHS) Foundation Trust Biomedical Research Centre.

**Table 12:** Summary of number of annotations generated from NLP applications in the SLaM BRC Case Register*

Application name	Total number of instances generated	Number of patients with at least one instance generated
MMSE	107 384	24 705
Diagnosis	615 237	78 851
Smoking	670 053	52 700
Education	181 905	51 665
Medication (selected)†		
Olanzapine	371 754	25 697
Citalopram	144 072	24 363
Mirtazapine	135 309	23 710
Risperidone	240 068	22 046
Zopiclone	129 488	20 712
Diazepam	129 409	17 841
Lorazepam	119 357	15 637
Fluoxetine	96 258	15 527
Sertraline	95 381	13 600
Promethazine	112 256	12 861
Clonazepam	111 279	9679
Quetiapine	98 509	9503
Aripiprazole	90 866	8737
Haloperidol	53 936	7591
Amisulpride	58 751	6759
Methadone	128 132	6385
Flupentixol	25 576	5248
Clozapine	111 170	4364
Zuclopenthixol	18 099	3093

*The CRIS database is updated every 24 h, so numbers are dynamic and displayed for illustrative purposes. NLP application run dates as follows: MMSE (24 June 2014), diagnosis (20 June 2014), smoking (17 July 2014), education (30 June 2014), medication (16 June 2014). †Most frequent 15 agents plus those evaluated in table 11.

CRIS, Clinical Record Interactive Search; MMSE, Mini-Mental State Examination; NLP, natural language processing; SLaM BRC, South London and Maudsley National Health Service (NHS) Foundation Trust Biomedical Research Centre.

### Findings to DATE

The SLaM BRC Case Register has been used for a wide range of research projects to date, as well as for key service evaluation and audit projects, and over 50 publications have arisen. Large-scale outcome studies supported by CRIS data have included those of residential mobility and of homelessness among inpatients on mental health wards.^15^ ^16^ Evaluations of service interventions and other quality markers were also studied,^35^ ^36^ and investigations are increasingly focusing on early symptoms and treatment pathways in psychosis.^37^ ^38^ Keyword search functionality recently supported a large historic cohort study of service use and abuse experiences of trafficked people in contact with secondary mental health services.^39^

A particularly prominent theme has been the investigation of mortality and physical health outcomes in people with mental disorders. Initial reports highlighted the raised mortality and lower life expectancy of people in the most common disorder groups.^40–43^ More studies were carried out to attempt to profile those most at risk, which have indicated that disability and environmental circumstances appear to be more important than symptoms.^44^ ^45^ This was supported by a study showing that, in those who received specific structured risk assessments, clinician-perceived risk of self-neglect was a strong and independent predictor of mortality, whereas clinician-perceived risks of suicide and/or violence were not predictive.^46^ In terms of mortality predictors in specific patient groups, the impact of psychiatric comorbidity and psychological health on all-cause and cause-specific mortality in opioid use disorder has been evaluated, highlighting the importance of personality disorder and comorbid alcohol use disorder.^43^ Similarly, the importance of alcohol and drug use, physical illness, and functional impairment as predictors of mortality in individuals with personality disorder has been demonstrated, a group with and life expectancies at birth reduced by 17–19 years compared with the general population in England and Wales.^47^ ^48^ Mortality outcomes have been further evaluated in studies of cognitive impairment and delirium in older adults.^34^ ^49^

Studies of pharmacotherapy profiles have continued investigations into mortality as an outcome, most notably in a report identifying a marked reduction in people using clozapine, not explained by a range of potential confounders including service use.^32^ Another study found that atypical antipsychotic agents were not associated with higher mortality in people with vascular dementia.^33^ Further work will examine antipsychotic polypharmacy in more detail, following recent successful development of algorithms to capture this.^24^ As described earlier, utilising the keyword search functionality in CRIS, exposure to non-pharmacological agents such as khat was investigated,^14^ and a large series of cases with suspected neuroleptic malignant syndrome were successfully identified which allowed a matched case–control study of antipsychotic exposures potentially responsible.^12^ ^13^ The association between antidepressant use and risk of mania and bipolar disorder has also recently been investigated,^50^ as has antipsychotic use in children and adolescents with autistic spectrum disorder.^51^ Finally, the potential to use extensive routine data to monitor treatment response was exemplified in a recent study of people receiving acetylcholinesterase inhibitor treatments for Alzheimer's disease in which trajectories of cognitive function were plotted before and after treatment initiation in order to identify predictors of ‘response’—to our knowledge, the largest and most extensive cohort of its kind.^17^

Recent developments which are likely to generate substantial future output include the assembly of one of the largest cohorts to date of women with severe mental disorder who are followed from preconception and pregnancy to investigate medication use in relation to maternal and fetal outcomes.^52^ Supplementing CRIS-derived outcomes to large clinical research samples with genetic profiling has also begun to generate novel output, for example, indicating that a well-recognised genetic risk factor for schizophrenia may also be a risk factor for worse clinical outcomes after diagnosis.^53^ NLP applications have recently been extended to cover a range of affective and psychotic symptoms, allowing much more detailed phenotyping of large samples than a diagnosis alone provides,^54^ ^55^ and a range of adverse drug events have also recently been successfully captured.^56^

## Discussion

Currently, the SLaM BRC Case Register contains over 250 000 patient records and we believe it is the largest mental health data resource of its kind (ie, derived from the full EHRs for mental healthcare services). Since its original description, the database has nearly doubled in numbers of patients represented, but more importantly there have been key developments in the infrastructure to expand further the scale and depth of information available for research.^7^ These developments have been primarily in NLP and linkage with external data sets.

### Strengths and limitations of NLP

NLP is being applied increasingly to extract information from medical records, including applications for the detection of specific adverse drug events and other health events such as falls and nosocomial infections,^57–59^ as well as use to identify obesity status and obesity-related diseases.^60^ ^61^ Furthermore, mining patient electronic medical records has been found to be useful for detecting patterns in patient care and patient treatment habits.^62^ ^63^ Statistical text mining has been used to determine if patients suffer from comorbidities related to smoking, as well as detecting fall-related injuries, and regular expressions have been used to extract blood pressure values from progress notes.^64–66^ NLP has been useful for extracting medical information such as principal diagnosis, information related to employment and medication use from clinical narratives.^64^ ^67^ ^68^ This has led to a better understanding of the conditions patients face and potential interventions.^69^ Manual chart review for annotation has been used extensively and when appropriate rigour is applied, the information extracted is very reliable and is often used as the reference standard to evaluate IE systems. Although the potential of NLP in mental health research was recognised in 1992, there have been few applications in clinical records from this specialty beyond those used for de-identification purposes.^70^ However, progress is being made, including US studies using NLP to determine depression outcome, and adverse drug reactions, and characterisation of diagnostic profiles.^71–73^

Considering performances of NLP IE applications applied to clinical text, one study developed an NLP system for classifying patients with 15 comorbidity states for diseases related to obesity, found that the automated system performed well against manual expert rule-based systems, and concluded that even a relatively complex task was possible for an automated system on the basis of F-measures ranging from 0.48 for gastro-oesophageal reflux disease as a comorbidity to 0.96 for depression, and an overall system F-value of 0.60.^74^ Another study evaluated automatic ascertainment of smoking status in 502 de-identified medical discharge records with 11 groups producing annotations and F-measures varying from 0.33 to 0.70 for current smoking status and 0.44 to 0.76 for past smoking.^75^ F-measures for our applications were therefore relatively favourable. On the other hand, an application to identify and extract a patient’s smoking status from clinical narrative text from Spanish outpatient records, evaluated against manual annotations, cited precision and recall statistics for a smoker versus non-smoker classification of 85% and 90%, respectively, and those for a current versus past smoker classification as 91% and 94%.^76^ In our application, we achieved comparable precision but lower recall.^31^

Preliminary studies ascertaining postoperative complications using NLP have been cited as yielding encouraging results.^77^ ^78^ For example, in a recently conducted pilot study of statistical NLP for identifying cases of deep vein thrombosis (DVT) and pulmonary embolism (PE) from free-text electronic narrative radiology reports, the positive predictive value and sensitivity for DVT were 89% and 80%, respectively, and those for PE were 84% and 79%.^79^ Another NLP application developed to ascertain weekly warfarin doses reported findings of 90.8% precision and 99.7% recall, and a broader medication-ascertaining application achieved 86% precision and 77% recall.^68^ ^80^ In our own data, an evaluation of the NLP diagnosis application yielded a precision of 99% and a recall of 98% for vascular dementia, and our evaluations of the pharmacotherapy application found over 90% precision and recall for clozapine, although higher accuracy may be due to the combined use of structured data. It should be borne in mind that performances for one diagnosis or medication cannot be assumed to generalise to others, so it is still CRIS policy to advise de novo evaluation of application performance in studies investigating previously unevaluated entities. This is particularly pertinent to investigating antipsychotic medication prescribing, which is frequently preceded by clinical discussions and possibly tests (ie, clozapine); therefore, the presence of multiple annotations may not be reflective of current prescribing.

As displayed in table 12, the development of NLP IE applications to date has resulted in a very substantial expansion in data fields available for analysis within the SLaM BRC Case Register and in the ability to construct longitudinal data sets with repeated measures (as illustrated for MMSE score trajectories before and after initiation of dementia treatment).^17^ With increasing use of EHRs, we believe that NLP techniques have an important role to play, whether derived metadata are to be used for research or to enhance the quality of the clinical record. This is particularly pertinent for mental health records where text fields are often substantial and contain some of the most important clinical information. However, although its potential is substantial, it is important to bear in mind that there may be limits in the usefulness of NLP in EHR-sourced data resources, because of the high degree of variability in clinical text. As well as the well-recognised challenges of non-grammatical sentences, misspellings, idiosyncratic abbreviations and jargon, there are more complex issues to deal with such as the establishment of temporality (eg, timing of events described in long case summaries), the classification of documents and within-document text domains (eg, sections of the history or mental state assessment), and the development of standard ontologies, not to mention the challenges of translation and harmonisation across languages. An important decision in NLP application development at the outset is whether near-perfect performance is required at an individual level, or whether a lower performance probabilistic approach might be appropriate. The latter may be sufficient for analyses to be carried out over large samples, but the former is likely to be required if the application is then to be used for clinical decision support.

### Strengths and limitations of data linkages

As well as NLP applications, we were also able to expand the depth of information in this mental health case register through linkages with external data, including mortality, cancer and hospitalisation, with a primary care linkage recently developed and a linkage with education records fully approved and about to be implemented. Data linkage has been used in a variety of registers to enhance research questions. For example, nationwide data from the Icelandic Medicines Registry and the Database of National Scholastic Examinations were linked to study associations between drug treatment of attention deficit/hyperactivity disorder and academic performance.^81^ In Sweden, acute myocardial infarction episodes were linked with routinely collected data on hospital discharges, mental health and mortality.^82^ UK general practice data have been linked to national mortality, hospitalisation and disease register data at an individual level, and to census-derived socioeconomic data at a small area level.^83^ The Western Australian e-cohort of half a million children included data cross-linked across a number of administrative registers including education, mental healthcare, hospital discharges, midwives notifications, cancer registrations, a registry of births, deaths and marriages and emergency presentations.^4^

Techniques for achieving both valid and secure data linkages within a robust governance framework are becoming increasingly standardised. In the Western Australian system, in order to protect privacy, linkage and analysis tasks are performed separately and linked data sets have identifiers removed before they are made available to researchers. Comparable procedures are followed in CRIS linkages. The data linkage process in Western Australia involves probabilistic methods to calculate the likelihood that two records belong to the same entity (person, family, event and location), whereas an important feature of the UK NHS is the NHS number, a unique reference for all patients, which we were able to use as the primary link for health-related information with CRIS data. Unique identifiers assigned at birth also exist in a number of other countries, including the unique citizen identifier, Civil Personal Registration number in Denmark covering prescription drug purchases, hospital inpatient, emergency and outpatient encounters, admissions to psychiatric hospitals, a range of disease-specific registries, primary care data and cause of death.^84^ In Taiwan, social insurance enumeration systems have been used to create the National Health Insurance Research Database which has high national coverage and includes data from social insurance, health information, census and education resources.^85^

Record linkages are particularly valuable when they enable the capture of exposure data from one source and outcome data from another source, and have enabled novel investigations such as those attained through linking conscription surveys in Sweden and Israel with healthcare registers. Databases utilising the northern European system of unique citizen number will still have particular value in the following respects: (1) where information is gained on the total population within a geographic or administrative area, and not only insured patients; (2) where the person identifier is used for wider purposes than healthcare allowing novel and informative linkages, as discussed. The development of these linkages for the SLaM BRC Case Register is thus comparable with current practice elsewhere; however, the depth of information on mental healthcare accessed by CRIS is, we believe, currently unique in scale and scope, which we hope will enable findings from larger national samples to be further investigated in greater depth at a local level. There are various limitations with data linkage. First of all, most of the data linked to CRIS have time limitations, and cannot be used to develop decision support applications, because they are not available in real time. Mismatched identifier variables also place limits on the linkage process, although we have found this to be rare for the NHS number.

### Collaborations

Work to date on the SLaM BRC Case Register has involved a number of welcomed collaborations, including those with other academic groups, both national and international, as well as with industry partners in pharmaceutical and biotech sectors. The authors particularly acknowledge the longstanding and fruitful collaboration with the University of Sheffield Department of Computer Science on the application of NLP techniques. The primary consideration with collaboration is the requirement (a component of the Case Register's ethics approval) that all data remain within the NHS firewall during analysis. In order to facilitate this, a dedicated office suite was set up in SLaM premises, the ‘BRC Nucleus’ to accommodate staff and visitors accessing Case Register data, although remote access, with appropriate security, is also possible. A second requirement is an appropriate affiliation with SLaM for those accessing the data, most usually taking the form of an honorary or substantive contract, or a ‘research passport’, but also covered on occasions by appropriate between-institution legal agreements as directed by the SLaM Caldicott Guardian—the statutory office overseeing the use of patient information in the NHS. All research projects using CRIS are considered and approved by a patient-led Oversight Committee, reporting to the Caldicott Guardian, as described in detail elsewhere.^8^ As well as considering the appropriateness of research proposals, the CRIS Oversight Committee also adjudicate on risks of de-anonymisation at the analysis planning stage and, if needed, in the preparation of findings for publication (eg, proof-reading papers reporting quoted text excerpts).

### Implications and challenges for future developments

Data derived from EHRs have huge potential to contribute to research and clinical care. Observational data are vital in healthcare-relevant research. As well as research into disease risk factors, incidence and prognosis, an important application of EHR-derived data is in providing ‘real-world’ information on response to routine clinical interventions (eg, recovery, adverse events) and, most importantly, predictors of response. The ascertainment of characteristics predicting good/poor intervention response supports ‘personalised medicine’. Compared with EHRs, randomised trials are insufficiently powered, even when combined, to detect predictors of response, and their samples are frequently highly selected—hence the need for large, generalisable data sets containing detailed information on routine clinical care. For example, the recently reported CRIS study of MMSE score trajectories before and after acetylcholinesterase inhibitor treatment initiation in dementia captured data on at least eight times more person-years of treatment from a single mental healthcare provider than all randomised controlled trial samples combined, as well as providing the added generalisability of ‘real-world’ data.^17^ EHR databases also potentially allow enhanced and more effectively targeted recruitment for randomised controlled trials and other intervention evaluations, in addition to permitting pretrial modelling and efficiency planning. Approach for research study participation is generally considered to require prior consent (ie, ‘opt in’), and a ‘Consent for Contact’ model for patient recruitment has been developed at SLaM.^86^

In the UK, EHRs are now near-ubiquitous in primary care and mental healthcare, and rapidly becoming so in acute care. However, realising their potential for clinical research depends heavily on the quality and nature of EHR data. In mental healthcare, applications have been very limited to date. In particular, although nearly all mental health services use EHRs, most clinically relevant information (eg, on symptoms, interventions, outcomes) is recorded in text and therefore not accessible for large-scale analyses to inform service planning, or for algorithms to support clinical decision-making. Given the very high individual and societal impact of disorders such as schizophrenia, bipolar disorder, depression and dementia, and the large mental healthcare sector, this data deficiency is a major limitation. For example, current national data on mental healthcare in the UK are principally available from three sources: (1) primary care data resources such as the Clinical Practice Research Datalink which covers approximately 5–10% of general practices;^87^ (2) HES;^88^ and (3) the Mental Health Minimum Data Set (MHMDS). However, each has key limitations. Primary care data do not contain information on mental health service interventions or sufficient information on the symptoms for which interventions are received and with which outcomes are evaluated. HES data are primarily used for identifying inpatient episodes and have limited data on interventions or outcomes beyond service receipt. The MHMDS covers mental healthcare more comprehensively; however, data are essentially restricted to service-level interventions (eg, pharmacotherapy is not recorded), and information on symptomatology and context for most patients is restricted to the relatively coarse Health of the Nation Outcome Scales.^89^

One solution for improving the structure of routine clinical data in the EHR would be to impose this structure at the point of data entry. However, the applicability of this approach depends on the willingness of clinical staff to input structured data; the accuracy of form completion; and on the extent to which the disorders, interventions and outcomes can be captured in pre-prepared scales. Our experience has been that imposition of structured fields in a clinical record is difficult to achieve, and even more so to sustain, at least within mental healthcare. Furthermore, although a structured field improves data accessibility, it does not necessarily render the data any more valid. Even in a clinical context where data have inherent structure (eg, blood pressure recordings following hypertension treatment), this approach has limitations and may fail to capture influential contextual factors (eg, suboptimal adherence to antihypertensive treatment, or ‘white coat hypertension’). Application of structure is particularly challenging in mental healthcare where interventions are primarily determined by qualitatively reported experiences (symptoms), where outcomes rely on tracking improvement or deterioration of the same constructs, and where some interventions themselves are not readily prestructured (eg, psychotherapeutic strategies). Although constructs such as medication sound amenable to imposed structure, this is limited in UK services because of the mixed prescribing between primary and mental healthcare. Structured recording of current medication outside a prescribing database is difficult to maintain with any accuracy because there is no clear gain for clinicians to enter medication receipt in a structured field compared with recording the same information in text. We have demonstrated that it is feasible to obtain at least some novel structured information from routine mental health records on a range of clinical indicators using NLP. The over-riding advantage of this approach is that no additional ‘data entry’ is required by clinical staff beyond what is normal practice. The validity of the approach has been demonstrated in a typical mental health service EHR at SLaM and it is reasonable to suppose at least some generalisability to other UK mental health services, given the relatively standardised nature of clinical assessments and national training in psychiatry. However, clearly cross-applicability is important to evaluate and in this respect it is advantageous that the CRIS application was successfully implemented in 2014 at four other mental health Trusts with comparable EHR systems (http://www.slam.nhs.uk/research/d-cris). Finally, as with all data derived from routine sources, it is important to bear in mind, when designing investigations, the reasons why information may or may not be recorded in clinical practice—including the incentives for recording within different clinical services or at different points on the healthcare pathway. For example, in early analyses using the application to ascertain current smoking status, it was found that missing data were relatively high unless the focus was on patients who had received at least a year's care from SLaM.^31^ Enhancing the structure of a record could be one answer, although better design and focusing of text fields may in the end be more acceptable.

A more generic challenge for the use of specialist healthcare data lies in the limited time ‘windows’ within which data are provided. Cohort studies using such data resources therefore need to take into account not only what data are available from the record but also the time periods within which they are available. These time periods also need to be carefully considered in relation to the question under investigation, since they are determined by discharge and/or re-referral, which clearly themselves are determined by factors such as recovery, engagement with services and out-migration from the catchment. Those patients on whom longest periods of follow-up are available are likely to be those who have more severe symptomatology (requiring longer periods of care), although they may also have more stable accommodation or support and thus less likelihood of out-migration. Data linkages can provide some means of addressing the problem—for example, national data on hospitalisation or mortality accrue regardless of a patient's contact or not with mental healthcare; however, these may be limited in depth of information, as described above.

A key challenge inherent with all use of healthcare data is how to ensure such data are appropriately and robustly protected and how to develop and to use anonymised clinical information in a way that is acceptable to the general public, and most importantly to patients. Such challenges incorporate not only a case register's data themselves but also procedures around data linkage where use of identifiers is required, although systems are increasingly becoming established which achieve data linkage in ways that effectively preserve anonymity. Data protection laws and practice vary internationally, but most do have some provision for the use of data without prior consent if these data are effectively anonymised and if important research cannot be carried out in any other way. It is also worth bearing in mind at the outset that few data sets can be claimed to be wholly anonymised. For example, even in the shallowest of administrative databases, a combination of age, gender and date/place of admission might well be sufficiently unique that it theoretically identifies a person. Technical solutions to anonymisation are therefore never sufficient on their own, but need to be accompanied by a governance structure which evaluates database use for any risk of compromising anonymity, as well as monitoring the appropriateness of the research being carried out, and of the people and agencies having data access. The coming years will bring many more opportunities for the use and linking of anonymised EHR data. It is clear that researchers, patients and the general public need to be engaged in ongoing conversations and collaborations to develop appropriate frameworks so as to maximise the use of such data in ways that maintain the trust of all parties. The SLaM BRC Case Register involved patients from the outset both in designing the security model and in leading ongoing oversight of data use and dissemination,^8^ thus ensuring that discussions about the future of EHR use (scientifically, and as a sociological question) effectively and meaningfully engage the stakeholders whose data have generated the resource in the first place.
