# Supplementary material for: Cohort profile of the South London and Maudsley NHS Foundation Trust Biomedical Research Centre (SLaM BRC) Case Register: current status and recent enhancement of an Electronic Mental Health Record-derived data resource
Source: BMJ Open. 2016 Mar 1;6(3):e008721. doi: 10.1136/bmjopen-2015-008721 (PMC4785292; doi:10.1136/bmjopen-2015-008721)
Supplement: Supplementary appendix [file bmjopen-2015-008721supp_appendix.pdf]

APPENDIX – CRIS technical architecture

SLaM clinical data sources are de-identified and fed to the CRIS SQL and FAST indices. The FAST index provides the search engine for the CRIS front end. Subsequent to initial CRIS development, the SLaM Clinical Data Linkage Service (CDLS; described below) has been set up in order to process and house linked external datasets, located with CRIS within the SLaM electronic firewall. What has been termed metaphorically as a 'demilitarised zone' (DMZ) has additionally been created within this firewall where biological data can be uploaded and analysed, providing higher flexibility in terms of software provision, but also the appropriate level of security to host linked clinical data from CRIS. This accompanies and is driven by the parallel development of SLaM Bioresource, a growing bank of clinical samples providing 'omics and other biological data on a large scale. The DMZ, and the high performance computer cluster housing this, also support large-scale meta-data generation using natural language processing (described below). Finally, there has been the need to integrate myhealthlocker, a recently developed portal at SLaM where patients can, amongst other things, access some parts of their clinical record (e.g. care plans) and directly enter information (e.g. patient reported outcome measures) to PJS and thus to CRIS (http://www.slam.nhs.uk/patients-and-carers/patient-information/myhealthlocker).

The initial version of CRIS, previously described, consisted solely of the FAST index.[7] In response to an early need for greater flexibility in querying and retrieving data, a second, relational database version of the SLaM BRC Case Register was developed to operate alongside the original CRIS interface—SQLCRIS. A relational database is so-called because it is based on 'relations', which correspond to data tables or possible combinations of tables. Each relation in turn consists of a set of 'tuples' (rows) that map 'attributes' (column names)

to values of those attributes (data values). Using a suitable programming language, usually some form of Structured Query Language (SQL), database users may perform operations upon these relations, including the definition of new relations from existing ones, by means of relational operators (join, union, intersect, and so on). Commercially available SQL database programs include SQL Server (Microsoft), Oracle (Oracle Corporation) and DB2 (IBM). Such databases may typically be accessed both directly, through platform-specific interfaces such as Microsoft SQL Server Management Studio, and also indirectly, through interfaces to other software such as Stata (Stata Corp, College Station, TX) or SAS (SAS Institute Inc, Cary, NC).

SQLCRIS is a MS SQL Server 2008 database, whose tables are populated by ‘shredding’ the main XML index, so that the same ‘pipeline’ stages (including anonymisation) are used for both versions. There are around 100 tables, each of which corresponds to a ‘form’ in the electronic patient record. Full-text indexing is used for key open-text columns, improving performance on queries that reference these columns. Registered users are able only to issue queries on the main SQLCRIS schema, preventing possible data corruption. However, all users are able to use temporary tables and a separate schema is available for data uploads. Monitoring of user activity for oversight purposes is performed similarly to queries issued via CRIS: the CRIS administrator can return all queries issued by a given user. As yet, SQLCRIS is used directly by a minority of users; however, through investment in technical support and staffing, a much wider group of users are now routinely provided with data extracts taken from SQLCRIS, or use applications that interface with SQLCRIS.
